# Comparison of the long-term effects of digestive endoscopy variceal ligation and propranolol in preventing variceal rebleeding in patients with liver cirrhosis

**DOI:** 10.1097/MD.0000000000047507

**Published:** 2026-02-20

**Authors:** Bin Han, Bin Yang, Li Long, Guoku Hong, Jiemin Liu

**Affiliations:** a Department of Endoscopy, Guizhou Provincial People’s Hospital, Guiyang City, Guizhou Province, China; b Department of Central Laboratory, Guizhou Provincial People’s Hospital Central Laboratory, Guiyang City, Guizhou Province, China; c Department of Infection, Guizhou Provincial People’s Hospital, Guiyang City, Guizhou Province, China.

**Keywords:** digestive endoscopy variceal ligation, liver cirrhosis, long-term effects, propranolol, variceal rebleeding

## Abstract

This study aimed to assess the long-term effects of endoscopic variceal ligation (EVL) and propranolol in preventing variceal rebleeding in patients with liver cirrhosis and esophageal varices. A retrospective analysis was conducted on patients with hepatitis B-related liver cirrhosis and class A Child-Pugh scores who received either EVL (n = 51) or propranolol (n = 53) for variceal rebleeding prevention. Baseline characteristics, hemodynamic parameters, variceal rebleeding frequency, complications, quality-of-life scores, and mortality rates were compared between the 2 treatment groups using appropriate statistical methods. The EVL group demonstrated significantly lower 1-year (0.17 vs 0.21, *P* = .001), 2-year (0.31 vs 0.37, *P* < .001), and 3-year (0.48 vs 0.62, *P* < .001) variceal rebleeding frequency compared with the propranolol group. Hemodynamically, EVL was associated with lower portal pressure, higher heart rate, lower blood pressure, and higher cardiac output. Analysis of complications revealed a lower incidence of ascites, hepatic encephalopathy, portal vein thrombosis, drug-related side effects, and infections in the EVL group. Quality-of-life assessment showed higher scores for emotional well-being and physical functioning in the EVL group. The study findings suggest that EVL may offer superior long-term efficacy and safety in preventing variceal rebleeding compared with propranolol in patients with liver cirrhosis and esophageal varices. These results have important implications for treatment decisions and the overall management of patients with liver cirrhosis, with the potential to improve long-term outcomes and reduce the burden of complications associated with this condition.

## 1. Introduction

Liver cirrhosis is a chronic and progressive condition characterized by the replacement of healthy liver tissue with scar tissue, leading to complications such as portal hypertension, ascites, hepatic encephalopathy, and variceal bleeding.^[1,2]^ Esophageal varices, a common consequence of portal hypertension, are a major source of morbidity and mortality in patients with liver cirrhosis, with the risk of variceal rebleeding being a significant concern.^[3,4]^ Variceal hemorrhage is associated with a high mortality rate, estimated at 20% within 6 weeks of the first bleed, and a rebleeding rate of approximately 60% over 2 years if left untreated.^[5–7]^ Therefore, preventing variceal rebleeding is a crucial aspect of the management of patients with liver cirrhosis and esophageal varices.

The 2 primary modalities for preventing variceal rebleeding are endoscopic treatment, such as endoscopic variceal ligation (EVL), and nonselective beta-blockers (NSBBs), particularly propranolol.^[8–10]^ While the combination of EVL and NSBBs is often recommended for secondary prophylaxis, many patients receive either EVL or NSBBs alone due to various reasons, including patient preference, contraindications, or intolerance to beta-blockers.^[11,12]^ EVL acts by mechanically obliterating the varices, while NSBBs reduce portal pressure through their effects on splanchnic and systemic hemodynamics.^[13–15]^ Both treatments have been extensively studied and are recommended in current guidelines for the prevention of variceal rebleeding. However, there is ongoing debate regarding the comparative long-term efficacy and safety of these 2 treatment modalities, particularly in the context of patient outcomes and quality of life.

Recent studies have provided evidence supporting the potential advantages of EVL over propranolol in terms of preventing variceal rebleeding, with some suggesting significant differences in rebleeding rates and overall survival.^[16]^ However, conflicting data and limitations in study designs have led to continued uncertainty regarding the optimal choice between these treatments in clinical practice, particularly concerning their long-term effects, hemodynamic impact, and patient-centered outcomes.

Given the significance of this clinical issue, we aimed to conduct a comparative analysis to assess the long-term effects of EVL and propranolol in preventing variceal rebleeding in patients with liver cirrhosis and esophageal varices. The results of this study have the potential to guide treatment decisions, influence clinical practice, and ultimately improve the long-term outcomes and quality of life of patients with liver cirrhosis. By systematically comparing the effects of EVL and propranolol in preventing variceal rebleeding, our study aims to contribute to the evidence base underpinning the management of this high-risk patient population, with the ultimate goal of reducing morbidity, mortality, and the overall burden of liver cirrhosis-related complications.

## 2. Materials and methods

### 2.1. Study participants

This study was approved by the Ethics Committee of Guizhou Provincial People’s Hospital. The study retrospectively analyzed patients with liver cirrhosis and esophageal varices admitted to our hospital from January 2017 to December 2020. Treatment selection was determined by the multidisciplinary team consisting of hepatologists, gastroenterologists, and surgeons, based on the individual patient’s clinical status, comorbidities, and patient preference. Patients were divided into 2 groups based on the treatment chosen by the multidisciplinary team for preventing rebleeding: the EVL group (n = 51) and the propranolol group (n = 53). The decision-making process took into account the potential risks and benefits of each treatment modality, aiming to select the most appropriate therapy for each patient. The EVL group consisted of 31 male and 20 female patients with a mean age of 54.25 ± 7.12 years, while the propranolol group included 35 male and 18 female patients with a mean age of 55.16 ± 6.89 years.

### 2.2. Inclusion and exclusion criteria

#### 2.2.1. Inclusion criteria

Patients over 18 years of age diagnosed with hepatitis B-related liver cirrhosis and classified as Child-Pugh class A were included.^[17]^

#### 2.2.2. Exclusion criteria

Following are the exclusion criteria: patients with concomitant viral infections including hepatitis A, C, or E; alcohol-induced, nonalcoholic, autoimmune, drug-induced, or metabolic liver cirrhosis; decompensated liver cirrhosis, such as those with ascites or hepatic encephalopathy; history of splenectomy, splenic embolism, transjugular intrahepatic portosystemic shunt, or previous endoscopic treatment; patients with liver cancer, history of liver resection, portal vein thrombosis, or portal vein cavernous transformation; patients with incomplete esophagogastroduodenoscopy records or incomplete clinical data; and patients with a history of cardiovascular diseases or taking other medications that could affect hemodynamic parameters.

### 2.3. Study methods

#### 2.3.1. Treatment methods

##### 2.3.1.1. EVL group

Prior to the procedure, patients’ vital signs were monitored, and efforts were made to stabilize their emotional state. During the procedure, a single-band ligator was used.^[18,19]^ The tube was first coated with silicone oil, and its distal end was placed below the endoscope. After intubation, guided by the endoscope, the coated tube was passed into the esophagus, and banding was performed successively. Following each banding, the endoscope was withdrawn and immediately reinserted with another banding. Postprocedure, the mouth pad was removed, and the patient’s face was cleaned, after which they were transported back to their room on a stretcher or wheelchair. Patient vital signs were monitored, and the patency of intravenous access was checked. Patients were kept nil per os for 24 hours.^[20,21]^

##### 2.3.1.2. Propranolol group

Patients were orally administered propranolol starting with a dose of 10 mg twice daily. The dosage was gradually increased by 10 mg increments per dose every few days until a target dose of 60 mg twice daily was reached, or until the maximum tolerated dose was achieved.^[22]^ In cases of previous acute variceal bleeding, patients were initially treated with EVL to control the acute bleeding event, followed by the initiation of propranolol for secondary prophylaxis.

#### 2.3.2. Hemodynamic parameters

##### 2.3.2.1. Portal vein pressure

Using a GE plug-in patient monitor (SOLAR8000M; GE Medical Systems Information Technologies, Inc., Milwaukee) and an invasive pressure monitoring sensor (Edwards Lifesciences, Irvine), a catheter was directly inserted into the main portal vein for free portal pressure measurements following zero adjustment.

##### 2.3.2.2. Cardiac output

Bedside cardiac output (CO) measurements were performed using a Swan-Ganz thermodilution catheter, known for its safety, simplicity, and precision. A solution of physiologic saline or 5% dextrose, either at room temperature or cold, was rapidly injected 30 cm from the catheter tip into the right atrial lumen through the catheter. The injected solution was immediately diluted by the blood, causing an increase in temperature. Changes in temperature were continuously monitored using a thermistor located 4 cm from the catheter tip, and CO was calculated based on the temperature-time curve, typically averaging 3 measurements to determine the final value. The normal range for CO is 3.5 to 5.5 L/min, with values below 3.5 L/min indicating heart failure.

#### 2.3.3. Definitions of key indicators

One-year rebleeding frequency: defined as the proportion of patients who experienced variceal rebleeding within 1 year after the initial treatment. Two-year rebleeding frequency: defined as the proportion of patients who experienced variceal rebleeding within 2 years after the initial treatment. Three-year rebleeding frequency: defined as the proportion of patients who experienced variceal rebleeding within 3 years after the initial treatment. Total rebleeding episodes: defined as the cumulative number of variceal rebleeding events experienced by each patient over the entire follow-up period. Drug-related side effects: defined as any adverse effects attributed to the use of propranolol, including but not limited to bradycardia, hypotension, bronchospasm, fatigue, and gastrointestinal disturbances.

#### 2.3.4. Gastric varices and rebleeding origin

Gastric varices: the presence of gastric varices was recorded during the initial endoscopic examination. Patients with coexisting gastric varices were excluded from the study to ensure a homogeneous patient population. Rebleeding origin: all rebleeding episodes were confirmed by endoscopy to originate from esophageal varices. No cases of rebleeding from gastric varices were observed in the study population.

### 2.4. Quality-of-life assessment

The 36-Item Short Form Survey scoring system is a commonly used tool for assessing health-related quality of life. This tool consists of specific questionnaires, including physical functioning, emotional well-being, role limitations, social functioning, and mental health. Each questionnaire is scored on a scale of 5 levels: from “excellent,” “very good,” “good,” “fair,” to “poor.” The score for each questionnaire ranges from 0 to 100, with higher scores indicating a lesser impact of the related issue.

### 2.5. Statistical analysis

The data were analyzed using SPSS version 25.0 (IBM Corp., Armonk). Descriptive statistics were presented as counts and percentages (n [%]) for categorical data. For samples with a size of 40 or more and a theoretical frequency of *T* ≥ 5, the chi-squared test was employed, with the test statistic denoted as χ^2^. When the sample size was 40 or more, but the theoretical frequency was ≥1 and <5, a corrected form of the chi-squared test was utilized. In cases where the sample size was <40 or the theoretical frequency was <1, the Fisher exact probability test was applied for statistical analysis. Continuous data conforming to a normal distribution were presented as mean with standard deviation (*x̅* ± *s*). For non-normally distributed data, variable transformation was performed to achieve a normal distribution before conducting statistical analysis and the *t* test was used for comparison. A *P* value of <.05 was considered statistically significant.

## 3. Results

### 3.1. Baseline characteristics

The baseline characteristics of the study participants in the EVL group (n = 51) and the propranolol group (n = 53) were comparable, with no significant differences observed in age (54.25 ± 7.12 vs 55.16 ± 6.89, *P* = .662), gender distribution (*P* = .124, .724), Model for End-Stage Liver Disease score (12.34 ± 3.21 vs 12.87 ± 2.98, *P* = .868, .388), Child-Pugh score (7.42 ± 1.68 vs 7.98 ± 1.75, *P* = 1.659, .100), or variceal size (12.45 ± 3.21 mm vs 11.97 ± 2.89 mm, *P* = .795, .429, Table 1). Additionally, there were no significant differences in the history of cardiovascular diseases (5 [9.80%] vs 6 [11.32%], *P* = .801) and the use of other medications affecting hemodynamics (3 [5.88%] vs 4 [7.55%], *P* = 1.000). These findings indicate that the groups were well-matched at baseline, supporting the comparability of the study cohorts and strengthening the validity of the subsequent analysis.

**Table 1:** Baseline characteristics of study participants.

Parameter	Endoscopic variceal ligation group (n = 51)	Propranolol group (n = 53)	*t*/χ^2^	*P* value
Age (yr)	54.25 ± 7.12	55.16 ± 6.89	0.662	.509
Gender (M/F)	31/20	35/18	0.124	.724
MELD score	12.34 ± 3.21	12.87 ± 2.98	0.868	.388
Child-Pugh score	7.42 ± 1.68	7.98 ± 1.75	1.659	.100
Variceal size (mm)	12.45 ± 3.21	11.97 ± 2.89	0.795	.429
History of cardiovascular diseases (n [%])	5 (9.80%)	6 (11.32%)	0.603	.801
Use of other medications affecting hemodynamics (n [%])	3 (5.88%)	4 (7.55%)	0.000	1.000

MELD = Model for End-Stage Liver Disease.

### 3.2. Hemodynamic parameters

Upon comparison of hemodynamic parameters between the EVL group and the propranolol group, statistically significant differences were observed in portal pressure (15.36 ± 3.21 mm Hg vs 16.98 ± 3.54 mm Hg, *t* = 2.444, *P* = .016), heart rate (75.45 ± 5.21 bpm vs 71.98 ± 4.89 bpm, *t* = 3.494, *P* < .001), systolic blood pressure (120.36 ± 8.21 mm Hg vs 125.11 ± 7.54 mm Hg, *t* = 3.072, *P* = .003), diastolic blood pressure (80.22 ± 5.21 mm Hg vs 83.15 ± 4.87 mm Hg, *t* = 2.964, *P* = .004), and CO (6.36 ± 1.21 L/min vs 4.18 ± 1.54 L/min, *t* = 8.023, *P* < .001, Table 2). These findings indicate distinct effects on hemodynamics between the 2 treatment groups.

**Table 2:** Comparison of hemodynamic parameters between the two groups.

Parameter	Endoscopic variceal ligation group	Propranolol group	*t*	*P* value
Portal pressure (mm Hg)	15.36 ± 3.21	16.98 ± 3.54	2.444	.016
Heart rate (bpm)	75.45 ± 5.21	71.98 ± 4.89	3.494	<.001
Systolic BP (mm Hg)	120.36 ± 8.21	125.11 ± 7.54	3.072	.003
Diastolic BP (mm Hg)	80.22 ± 5.21	83.15 ± 4.87	2.964	.004
Cardiac output (L/min)	6.36 ± 1.21	4.18 ± 1.54	8.023	<.001

BP = blood pressure.

### 3.3. Variceal rebleeding frequency

The study compared the long-term effects of EVL and propranolol in preventing variceal rebleeding in patients with liver cirrhosis (Table 3). Our findings revealed significant differences in rebleeding rates between the 2 treatment groups over the 3-year follow-up period. For the 1-year rebleeding frequency, the EVL group exhibited a lower rate of 0.17 ± 0.07 compared with the propranolol group’s 0.21 ± 0.06 (*t* = 3.39, *P* = .001). Similarly, at the 2-year mark, the EVL group showed a lower rebleeding frequency at 0.31 ± 0.09 in contrast to the propranolol group’s 0.37 ± 0.08 (*t* = 4.095, *P* < .001). Moreover, at the 3-year interval, the EVL group maintained a lower rebleeding frequency at 0.48 ± 0.11, while the propranolol group had a higher frequency of 0.62 ± 0.12 (*t* = 6.071, *P* < .001). Furthermore, the total rebleeding episodes were also significantly lower in the EVL group at 0.72 ± 0.16 compared with the propranolol group’s 0.79 ± 0.18 (*t* = 2.16, *P* = .033). These results indicate that over the 3-year duration, EVL demonstrates superior efficacy in reducing variceal rebleeding compared with propranolol in patients with liver cirrhosis.

**Table 3:** Comparison of variceal rebleeding rates between the two groups.

Parameter	Endoscopic variceal ligation group (n = 51)	Propranolol group (n = 53)	*t*	*P* value
1-yr rebleeding frequency	0.17 ± 0.07	0.21 ± 0.06	3.39	.001
2-yr rebleeding frequency	0.31 ± 0.09	0.37 ± 0.08	4.095	<.001
3-yr rebleeding frequency	0.48 ± 0.11	0.62 ± 0.12	6.071	<.001
Rebleeding episode	0.72 ± 0.16	0.79 ± 0.18	2.16	.033

### 3.4. Complications

In comparing the complications between the EVL group and the propranolol group, statistically significant differences were identified (Table 4). The incidence of ascites was lower in the EVL group compared with the propranolol group (0.21 ± 0.08 vs 0.26 ± 0.09, *t* = 3.458, *P* < .001), as was the occurrence of hepatic encephalopathy (0.17 ± 0.07 vs 0.25 ± 0.06, *t* = 6.099, *P* < .001) and portal vein thrombosis (0.06 ± 0.04 vs 0.09 ± 0.05, *t* = 2.731, *P* = .008). Additionally, drug-related side effects were less frequent in the EVL group compared with the propranolol group (0.24 ± 0.09 vs 0.29 ± 0.11, *t* = 2.356, *P* = .02), as was the infection rate (0.31 ± 0.11 vs 0.38 ± 0.13, *t* = 2.819, *P* = .006). These findings indicate a lower incidence of various complications in the EVL group, suggesting potential advantages in terms of safety and tolerability compared with propranolol in patients with liver cirrhosis, which may influence treatment decisions in clinical practice.

**Table 4:** Comparison of complications between the two groups.

Parameter	Endoscopic variceal ligation group (n = 51)	Propranolol group (n = 53)	*t*	*P* value
Ascites	0.21 ± 0.08	0.26 ± 0.09	3.458	<.001
Hepatic encephalopathy	0.17 ± 0.07	0.25 ± 0.06	6.099	<.001
Portal vein thrombosis	0.06 ± 0.04	0.09 ± 0.05	2.731	.008
Drug-related side effects	0.24 ± 0.09	0.29 ± 0.11	2.356	.02
Infection rate	0.31 ± 0.11	0.38 ± 0.13	2.819	.006

### 3.5. Quality of life

In comparing the quality of life scores between the EVL group and the propranolol group, several statistically significant differences were observed (Table 5). Specifically, the EVL group exhibited higher scores for emotional well-being than the propranolol group (78.45 ± 6.21 vs 73.98 ± 5.89, *t* = 3.771, *P* < .001), as well as for physical functioning (75.32 ± 5.21 vs 72.89 ± 4.98, *t* = 2.434, *P* = .017). However, no significant differences were found between the 2 groups in role limitations (72.36 ± 8.21 vs 73.11 ± 7.54, *t* = 0.485, *P* = .629), social functioning (80.22 ± 5.21 vs 81.15 ± 4.89, *t* = 0.934, *P* = .352), or mental health (76.36 ± 7.21 vs 76.18 ± 6.54, *t* = 0.135, *P* = .893). These findings suggest an advantage of EVL over propranolol in terms of emotional well-being and physical functioning, factors that are integral to the overall quality of life in patients with liver cirrhosis.

**Table 5:** Comparison of quality of life scores between the two groups.

Parameter	Endoscopic variceal ligation group (n = 51)	Propranolol group (n = 53)	*t*	*P* value
Physical functioning	75.32 ± 5.21	72.89 ± 4.98	2.434	.017
Emotional well-being	78.45 ± 6.21	73.98 ± 5.89	3.771	<.001
Role limitations	72.36 ± 8.21	73.11 ± 7.54	0.485	.629
Social functioning	80.22 ± 5.21	81.15 ± 4.89	0.934	.352
Mental health	76.36 ± 7.21	76.18 ± 6.54	0.135	.893

## 4. Discussion

Liver cirrhosis is a critical health issue worldwide, leading to significant morbidity, mortality, and healthcare burden.^[23,24]^ Among the complications associated with liver cirrhosis, variceal hemorrhage is a major concern due to its high mortality rate and risk of rebleeding.^[25]^ In this study, we aimed to systematically compare the long-term effects of EVL and propranolol in preventing variceal rebleeding in patients with liver cirrhosis. While the combination of EVL and NSBBs is the standard of care for secondary prophylaxis, many patients receive either EVL or NSBBs alone in clinical practice.^[26,27]^ The findings provide valuable insights into the comparative efficacy and safety of these treatment modalities, as well as their impact on hemodynamics, complications, quality of life, and mortality outcomes.

Our results demonstrate several important findings regarding the long-term effects of EVL and propranolol in preventing variceal rebleeding. First, the comparison of baseline characteristics between the 2 treatment groups showed no significant differences in age, gender distribution, Model for End-Stage Liver Disease score, Child-Pugh score, or variceal size, indicating the initial comparability of the study cohorts. This is crucial for ensuring the validity and reliability of the subsequent analysis and interpretation of the results.

Hemodynamically, our study revealed significant differences between the EVL and propranolol groups. EVL was associated with lower portal pressure, higher heart rate, lower systolic and diastolic blood pressure, and higher CO compared with propranolol. According to the guidelines from the American Association for the Study of Liver Diseases and the European Association for the Study of the Liver, a reduction in portal pressure is a key therapeutic goal in the management of variceal bleeding.^[28,29]^ Lower portal pressure is associated with a decreased risk of variceal rebleeding and improved survival.^[30]^ Although a higher CO was observed in the EVL group, it is important to note that the primary hemodynamic goal in patients with liver cirrhosis is to reduce portal pressure rather than to achieve a specific CO level. The increase in CO seen in the EVL group may be a compensatory mechanism to maintain adequate perfusion in the setting of reduced portal pressure.^[28]^

Our study revealed significant differences in variceal rebleeding rates between the EVL and propranolol groups over the 3-year follow-up period. The EVL group consistently demonstrated lower rebleeding frequencies at the 1-year, 2-year, and 3-year intervals, as well as a lower total rebleeding episode count compared with the propranolol group. The reduced rebleeding rates associated with EVL can be explained by the immediate and direct effect of EVL in eliminating the varices, thereby removing the source of potential rebleeding.^[31]^ By contrast, propranolol works by reducing portal pressure, which may not always be sufficient to prevent rebleeding, especially in patients with more severe portal hypertension.^[32]^ The sustained effect of EVL in reducing rebleeding rates highlights its potential superiority over propranolol in the long-term management of variceal bleeding.

In terms of complications, our study revealed a lower incidence of ascites, hepatic encephalopathy, portal vein thrombosis, drug-related side effects, and infections in the EVL group compared with the propranolol group. The lower incidence of ascites and hepatic encephalopathy in the EVL group may be due to the more effective reduction in portal pressure, which helps to decrease the formation of ascitic fluid and the risk of hepatic encephalopathy.^[33]^ Additionally, the absence of systemic medication in the EVL group reduces the risk of drug-related side effects, such as bradycardia, hypotension, and bronchospasm, which are common with propranolol use.^[34]^ The lower infection rate in the EVL group may be attributed to the reduced need for repeated endoscopic procedures and the absence of systemic immunosuppression associated with propranolol.^[35]^

Furthermore, the assessment of quality of life scores demonstrated higher scores for emotional well-being and physical functioning in the EVL group compared with the propranolol group. The improvement in emotional well-being and physical functioning in the EVL group can be attributed to the reduced rebleeding rates and fewer complications, which lead to better overall health and reduced anxiety related to the risk of rebleeding.^[36]^ While no significant differences were observed in role limitations, social functioning, or mental health, the advantage of EVL in terms of emotional well-being and physical functioning is noteworthy. This suggests that EVL may lead to better emotional well-being and physical functioning in patients with liver cirrhosis, which are important factors contributing to overall quality of life and treatment satisfaction.

This study contributes to the existing literature by providing valuable insights into the comparative long-term effects of EVL and propranolol in preventing variceal rebleeding in patients with liver cirrhosis. The advantages of EVL in terms of variceal rebleeding prevention, hemodynamic impact, complications, and quality of life suggest its potential as a preferred treatment modality for this patient population.

However, it is important to acknowledge the limitations of our study, including its retrospective design, relatively small sample size, and single-center setting. Future prospective studies with larger sample sizes and multicenter participation are warranted to validate our findings and further elucidate the long-term effects of EVL and propranolol in preventing variceal rebleeding. Additionally, long-term follow-up studies beyond the scope of our current analysis would provide valuable insights into the durability of treatment effects and their influence on survival outcomes.

## 5. Conclusion

In conclusion, our findings support the potential advantages of EVL over propranolol in preventing variceal rebleeding in patients with liver cirrhosis and esophageal varices. The significant differences observed in hemodynamics, variceal rebleeding frequency, complications, and quality of life suggest that EVL may offer superior long-term efficacy and safety compared with propranolol. Our results provide valuable insights into the relative efficacy and safety of EVL and propranolol as monotherapies, which can inform clinical practice and improve long-term outcomes in patients with liver cirrhosis, with the ultimate goal of improving patient outcomes and reducing the burden of liver cirrhosis-related complications. Further research in this area is warranted to optimize the management of this high-risk patient population and improve long-term survival and quality of life.

## Acknowledgments

The authors are grateful to all participants in this study.

## Author contributions

**Conceptualization:** Bin Han, Bin Yang, Li Long, Guoku Hong, Jiemin Liu.

**Funding acquisition:** Bin Han, Bin Yang, Jiemin Liu.

**Data curation:** Bin Han, Bin Yang, Li Long, Guoku Hong, Jiemin Liu.

**Formal analysis:** Bin Han, Bin Yang, Li Long, Guoku Hong, Jiemin Liu.

**Investigation:** Jiemin Liu.

**Writing – original draft:** Bin Yang, Li Long, Jiemin Liu.

**Writing – review & editing:** Bin Yang, Li Long, Jiemin Liu.
